# Amifostine Analog, DRDE-30, Attenuates Bleomycin-Induced Pulmonary Fibrosis in Mice

**DOI:** 10.3389/fphar.2018.00394

**Published:** 2018-04-24

**Authors:** Aastha Arora, Vikas Bhuria, Puja P. Hazari, Uma Pathak, Sweta Mathur, Bal G. Roy, Rajat Sandhir, Ravi Soni, Bilikere S. Dwarakanath, Anant N. Bhatt

**Affiliations:** ^1^Institute of Nuclear Medicine & Allied Sciences, New Delhi, India; ^2^Department of Biochemistry, Panjab University, Chandigarh, India; ^3^University Hospital Tübingen, Tübingen, Germany; ^4^Synthetic Chemistry Division, Defence Research and Development Establishment, Gwalior, India

**Keywords:** lung inflammation, lung fibrosis, bleomycin, DRDE-30, Amifostine, micro-computed tomography

## Abstract

Bleomycin (BLM) is an effective curative option in the management of several malignancies including pleural effusions; but pulmonary toxicity, comprising of pneumonitis and fibrosis, poses challenge in its use as a front-line chemotherapeutic. Although Amifostine has been found to protect lungs from the toxic effects of radiation and BLM, its application is limited due to associated toxicity and unfavorable route of administration. Therefore, there is a need for selective, potent, and safe anti-fibrotic drugs. The current study was undertaken to assess the protective effects of DRDE-30, an analog of Amifostine, on BLM-induced lung injury in C57BL/6 mice. Whole body micro- computed tomography (CT) was used to non-invasively observe tissue damage, while broncheo-alveolar lavage fluid (BALF) and lung tissues were assessed for oxidative damage, inflammation and fibrosis. Changes in the lung density revealed by micro-CT suggested protection against BLM-induced lung injury by DRDE-30, which correlated well with changes in lung morphology and histopathology. DRDE-30 significantly blunted BLM-induced oxidative stress, inflammation and fibrosis in the lungs evidenced by reduced oxidative damage, endothelial barrier dysfunction, Myeloperoxidase (MPO) activity, pro-inflammatory cytokine release and protection of tissue architecture, that could be linked to enhanced anti-oxidant defense system and suppression of redox-sensitive pro-inflammatory signaling cascades. DRDE-30 decreased the BLM-induced augmentation in BALF TGF-β and lung hydroxyproline levels, as well as reduced the expression of the mesenchymal marker α-smooth muscle actin (α-SMA), suggesting the suppression of epithelial to mesenchymal transition (EMT) as one of its anti-fibrotic effects. The results demonstrate that the Amifostine analog, DRDE-30, ameliorates the oxidative injury and lung fibrosis induced by BLM and strengthen its potential use as an adjuvant in alleviating the side effects of BLM.

## Introduction

Idiopathic pulmonary fibrosis (IPF) is a progressive and debilitating interstitial lung disease (ILD), which is characterized by gradual scarring of normal lung tissue, causing respiratory distress. It causes considerable morbidity and mortality in humans, with the median survival time between 2–5 years following diagnosis (Hutchinson et al., 2015). IPF has a variable etiology, out of which exposure to silica/asbestos, smoking, genetic factors, infectious agents and anti-cancer drugs such as bleomycin are the factors of prominence (Zisman et al., 2005). The pathogenesis of IPF involves alveolar epithelial and endothelial cell damage, compensatory hyperplasia of type II pneumocytes, accumulation and proliferation of fibroblasts and myofibroblasts, and the subsequent deposition of extracellular matrix (ECM) proteins, such as fibronectin and collagen, causing stiffening of the lung tissue that progressively leads to aberrant/loss of lung function and respiratory failure (Selman et al., 2001; Phan, 2002). Despite having an immense effect on human health, there is still a dearth of approved treatment strategies that directly target the mechanisms of fibrosis, and no proven anti- fibrotic therapy has been effective in alleviating/reversing the fibrotic diseases (Paz and Shoenfeld, 2010). However, after large multicentre clinical trials, two oral treatment options: nintedanib (Ofev, inhibitor of PDGF/VEGF/FGF receptor tyrosine kinases) and pirfenidone (Esbriet, anti- inflammatory and anti-fibrotic agent) have recently been approved for treating mild to moderate cases of IPF, both causing only a partial reduction in the disease progression (reduce the decline in lung function, measured as Forced Vital Capacity) but no cure or regression of the disease (King et al., 2014; Richeldi et al., 2014). Hence, the identification of novel compounds and their preclinical evaluation in animal models of lung fibrosis presents an urgent need.

Intra-tracheal instillation of bleomycin (ITB) in rodents (mouse, rat, and hamster) is a well established, widely accepted and the most commonly used model of lung fibrosis since it closely resembles the human fibrotic lung disease (Williamson et al., 2015). The bleomycin model is quite simple and rapid to develop, is reliable and reproducible, and thus, fulfills all the important criteria which are expected out of a good animal model (Moeller et al., 2008; Mouratis and Aidinis, 2011). This model is characterized by acute lung injury and inflammation followed by progressive but resolvable pulmonary fibrosis (Izbicki et al., 2002). Occurrence of fibrosis as one of the major adverse effects of bleomycin in human chemotherapy forms the basis for the frequent use of bleomycin in developing animal models of pulmonary fibrosis. The mortality rate of patients with bleomycin-induced fibrosis has been reported to be approximately 3% of all patients treated with bleomycin (Levi et al., 1993). The unusual lung toxicity following intra-peritoneal injection of bleomycin has been linked to the higher concentration of the drug in the lung (Umezawa et al., 1967), as well as low levels of the bleomycin inactivating enzyme, bleomycin hydrolase, that critically influences the effects of this drug on different tissues (Sebti et al., 1989). Highly reactive oxygen and nitrogen species generated in the lung following bleomycin administration are at least partially responsible for its therapeutic, anti-neoplastic as well as toxic side effects. The main mechanism behind its anti-tumor effect is, however, at the level of DNA strand scission (Hay et al., 1991). Since ROS and inflammation are important contributing factors in bleomycin-induced pulmonary fibrosis, inhibiting these factors might help in curtailing the extent of the pulmonary infection. The activity of the intracellular antioxidant enzyme catalase has been linked with decreased lung fibrosis in a mouse model (Odajima et al., 2010). Additionally, *N*-acetylcysteine (NAC), an exogenous antioxidant, was demonstrated to dramatically decrease lung damage in the presence of TGF-β1 by reducing intracellular ROS production (Felton et al., 2009). Other molecules with anti-oxidant properties that have shown anti-fibrotic effect in the bleomycin model include Molsidomine (MOL, a potent vasodilator agent), Quercetin, Resveratrol and Berberine (an isoquinoline alkaloid; Akgedik et al., 2012; Chitra et al., 2013; Verma et al., 2013; Kilic et al., 2014). Recently, Thalidomide has also been shown to suppress bleomycin-induced pulmonary fibrosis through its anti-oxidative (through activation of thioredoxin reductase) and anti-inflammatory effects (Dong et al., 2017). Although the beneficial role of antioxidant enzymes and scavengers of free radicals in reducing the severity of bleomycin-induced lung injury has been demonstrated well in pre-clinical animal models, no established and regulatory approved drugs belonging to this class are available yet for clinical use.

The bleomycin-induced lung injury model has contributed immensely in identifying potential anti-fibrotic treatment options for IPF (preventive as well as therapeutic), many out of which have been suggested for clinical use. Compounds that have demonstrated anti-fibrotic potential in the bleomycin model belong to the following classes: Antioxidants (*N*-acetylcysteine, Melatonin, Quercetin, Curcumin), Angiotensin converting enzyme inhibitors (Captopril, Ramipril), Cytokines (IL-1β, IL-10, IL-18, CD-36, keratinocyte growth factor, hepatocyte growth factor), Cytokine blocking antibodies (TGF-β, TNF-α, IL-12, platelet derived growth factor, vascular endothelial growth factor), macrolide antibiotics (Azithromycin, Erythromycin, Clarythromycin), anticoagulants (Warfarin, heparin), corticosteroids (Prednisolone, Dexamethasone), Anti-inflammatory/immunomodulatory/immunosuppressive agents, chelating agents (D-Penicillamine, Pirfenidone), Endothelin receptor antagonists and metabolic pathway inhibitors (Moeller et al., 2008; Della Latta et al., 2015). However, only a small proportion of these ‘promising compounds’ were or are being tested in clinical trials, out of which only the recent clinical trials of NAC, pirfenidone and nintedanib gave hope to the patients of IPF. Unfortunately, the placebo-controlled PANTHER-IPF study revealed no positive effects of NAC on the study endpoints (FVC) and the results did not provide any support for the clinical use of NAC in the treatment of IPF (Martinez et al., 2014). However, two phase III trials have yielded positive outcomes so far, in the form of pirfenidone and nintedanib. The current understanding of the pathogenesis of IPF as an aberrant wound healing response suggests novel targets for investigational strategies like targeting extra cellular matrix deposition, use of tyrosine kinase antagonists, modulation of immune response and cell-based therapy (Totey, 2017; Varone et al., 2017). Further, since IPF is an age related disease paradigm, many age-related cellular abnormalities like telomere attrition, senescence, genomic instability and epigenetic alterations are being proposed as potential targets of interest for developing treatment strategies for IPF (Myllärniemi and Kaarteenaho, 2015; Mora et al., 2017; Tashiro et al., 2017; Povedano et al., 2018). Therefore, the need for developing potential protective compounds and/or identifying the ones from existing pharmacopeia continues.

One of the widely investigated compounds has been Amifostine (S-2[3-aminopropylamino] ethylphosphorothioic acid) (WR-2721). After dephosphorylation by membrane-bound alkaline phosphatase in tissues, WR-2721 gets converted into the cell permeable, bioactive free thiol form, WR-1065, that rapidly penetrates into cells, where the thiol groups act as free-radical scavengers and protect cells from oxidative stress induced damage. Amifostine provides broad spectrum cytoprotection to various normal tissues when administered prior to cytotoxic chemotherapy (alkylating agents, organoplatinums, anthracyclines) or radiation, without compromising the antitumor effect (Capizzi, 1996). Cytoprotective levels of Amifostine or its metabolites can be found in many organs, including the lungs, where it has been shown to protect against the toxic effects of bleomycin and radiation (Nici and Calabresi, 1999; Vujaskovic et al., 2002). Amifostine is generally well tolerated, although dose related adverse effects may occur transiently, the most clinically significant and dose limiting toxicity being hypotension. The utility of Amifostine, thus, is limited by its inherent systemic toxicity, tissue specificity due to differential level of alkaline phosphatase in different tissues, unfavorable routes of administration, high cost as well as a narrow therapeutic window.

In a similar quest of developing more potent, easily administrable and safer antidotes against the chemical warfare agent Sulfur Mustard (SM), various analogs of Amifostine were synthesized by Defence Research and Development Establishment (DRDE), Gwalior, India, by modifying the side chains; one of them was S-2 (2-aminoethylamino) ethyl propyl sulfide or DRDE-30 (Bhattacharya et al., 2001; Vijayaraghavan et al., 2001). DRDE-30, unlike Amifostine, is devoid of a phosphate group and does not require cleavage by alkaline phosphatase for activation, due to which it might demonstrate its protective effects even in tissues with low levels of the enzyme. Also, because of the presence of an alkyl group, DRDE-30 has enhanced lipophilicity and thus, better bioavailability than Amifostine (Kumar et al., 2010). DRDE-30 has been demonstrated to confer protection against percutaneously administered SM in mice, accompanied by restoration of the liver GSH levels as well as reduction in the DNA damage induced by SM, when given orally as well as intraperitoneally (Bhattacharya et al., 2001; Pathak et al., 2004; Kulkarni et al., 2006). DRDE-30 has also been shown to inhibit inflammation in carrageenan-induced paw inflammation model, where its efficacy was found to be comparable to the standard drug aspirin (Bhutia et al., 2010). Since the mechanisms underlying the toxicity of SM share a great deal of similarity with damage induction by bleomycin, including DNA damage and oxidative stress, it was hypothesized that DRDE-30 would confer protection against bleomycin-induced toxicity. Thus, the present studies were undertaken to investigate the effects of DRDE-30 on bleomycin-induced pulmonary fibrosis in mice.

## Materials and Methods

### Animals

Female, adult, 8–10 weeks old C57BL/6 mice, with an average weight of 20–25g were issued from the Experimental Animal Facility of INMAS, DRDO, Delhi, India. They were housed in groups of 6 (maximum) per cage (22 ± 2°C and 12–12 h/light- dark cycle) with free access to a standard laboratory rodent diet (Golden Feeds, Delhi, India) and water *ad libitum*. The animals were acclimatized for 1 week prior to the experiments. The study protocols were reviewed and approved by the Institute’s Committee on the Ethics of Animal Experiments before the experiments began (Institutional Ethical Committee Number: INM/IAEC/16/25).

### Mouse Model of Bleomycin-Induced Lung Injury

Mice were anesthetized with a combination of Ketamine (80 mg/kg body weight) and Xylazine (5 mg/kg body weight) and placed on an intubation stand facing upward at an angle of approximately 45° by using an elastic string carefully positioned under the animal’s front incisors. The tongue was gently pulled out with forceps and the trachea was intubated with a 22-G plastic sterile i.v. catheter. Bleomycin sulfate (available as 15U Bleocip injection, Cipla) at 100 mU, prepared in 30 μl of sterile saline was then slowly instilled through the catheter into the trachea.

### Treatment Allocation

DRDE-30 (200 mg/kg body weight) was prepared freshly at the time of administration by dissolving in sterile phosphate buffered saline (PBS). The injectable volume was 200 μl/mouse administered intra-peritoneally (IP) 30 min prior to intra-tracheal (IT) instillation of bleomycin.

**FIGURE 1:**
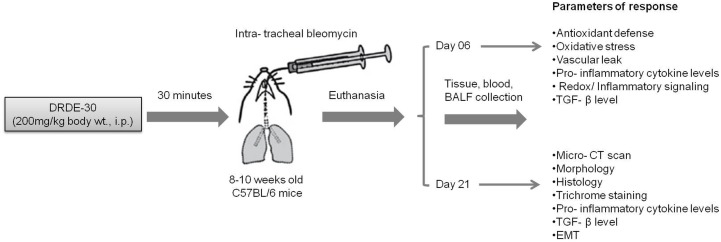
Schematic illustration of the experimental design of the study describing the treatment regimen, timing of sacrifice and the parameters studied.

Animals were randomized into 4 weight-matched experimental groups: (i) Control group, animals received IP PBS/IT saline; (ii) Bleomycin group, animals received IP PBS/ IT bleomycin; (iii) Drug (DRDE-30) alone group, animals received IP drug/ IT saline; (iv) Bleomycin plus drug group, animals received IP drug/IT bleomycin. Animals were monitored for morbidity and mortality till the completion of the study. A general scheme describing the experimental design is given in **Figure 1**.

### *In Vivo* Micro-Computed Tomography (CT) Analysis

Twenty one days after treatment, *in vivo* micro-CT analysis of the whole animal was performed for assessment of BLM-induced changes in lung density due to differential x-ray absorption. Animals were anesthetized with intraperitoneal injection of Ketamine (80 mg/kg body wt.) and Xylazine (5 mg/kg body wt.) and fixed in prone position. Micro-CT images were acquired in FLY mode on the trimodal GE-FLEX Triumph micro-PET/SPECT/CT Scanner (TriFoil Imaging, Northridge, CA, United States) using the following parameters: 75 kV; 170 μA; focal spot size: 50 μ; Magnification 2.0; FOV 59.2 mm; 512 projections, resulting in a total acquisition time of approximately 4 min. Resulting images were reconstructed and analyzed using AMIRA 4.1.1 software.

### Histological Assessment of Lung Injury

After 21 days of treatment, mice were sacrificed to surgically isolate the lungs for histological analysis. The lungs were instilled with neutral buffered formalin (10%) and then immersed in the fixative for 16–18 h at room temperature, embedded in paraffin, and sectioned at 5 μm thickness. After removing paraffin and rehydration, the lungs were stained with haematoxylin and eosin and observed under Olympus (IX51) microscope (Japan) for evaluating lung injury by bright field microscopy. A scoring system, called the Ashcroft score (Ashcroft et al., 1988), was used to grade the degree of lung injury. Six high power fields (HPFs) were scored per section, five sections were scored per mouse and 4 mice were scored per group. Finally, the total score was calculated for each animal.

### Masson’s Trichrome Staining

Paraffin-embedded, transverse lung sections (5 μm) were cut and stained using Masson’s trichrome stain (Sigma-Aldrich, Saint Louis, MO, United States) to identify the sites and extent of collagen deposition.

### Determination of Lung Hydroxyproline Content

Lung collagen at the end of 21 days was determined by estimating the amount of hydroxyproline present in the tissue sample using the Hydroxyproline Assay Kit (Sigma-Aldrich, Saint Louis, MO, United States) as per the manufacturer’s instructions. Briefly, lungs were cleared off the extraneous material and washed with PBS. 10mg of tissue was homogenized in 100 μl of ice cold Milli-Q water and transferred to a pressure-tight glass vial. Hundred microliter of 12N HCl was added to the vial, capped tightly, and hydrolyzed at 120°C for 3 h. The hydrolysate was then separated from the particulate matter by centrifugation. Twenty five microliter of sample was transferred to a 96-well plate and dried in a 60°C oven. Hundred microliter Chloramine-T solution was added to the wells and incubated at room temperature for 5 min, followed by incubation with 100 μl Ehrlich’s Solution for 90 min at 60°C. Absorbance was measured at 550 nm and hydroxyproline concentrations in the sample was calculated from the standard curve generated using known concentrations of trans-4-hydroxyl-L-proline (Sigma H5534). Results were expressed as micrograms of hydroxyproline per ml sample.

### Measurement of Lipid Peroxidation

Malondialdehyde (MDA) levels in the lungs, 06 days post treatment, were determined by measuring the absorbance of the colored product obtained from the reaction of thiobarbituric acid-reactive substances (TBARS) with 2-thiobarbituric acid (TBA), the TBARS-TBA adduct, according to the method of Buege and Aust (1978). MDA is a major representative of TBARS. After euthanizing the mice and lavaging the right lung, a portion of the superior lobe of the right lung was excised for performing the biochemical measurements and assessing the level of lipid peroxidation. The lung tissue was thoroughly rinsed in PBS, blotted dry and weighed before homogenizing. A 10% (w/v) tissue homogenate was prepared in chilled Tris- KCl buffer (10 mM Tris-HCl, 150 mM KCl, pH 7.4). Homogenates were spun in cold centrifuge at 10,000 g for 30 min at 4°C. One volume of the homogenate and 2 volumes of the Beuge-Aust reagent (0.37% w/v TBA and 15% w/v tri-chloro acetic acid, TCA in 0.25N HCl) were combined in a screw capped centrifuge tube, vortexed and heated in a boiling water bath for 15 min. After cooling the solution and removal of the precipitate, the absorbance of the clear supernatant was recorded spectrophotometrically at 532 nm against a sample blank containing reagents but no sample. Quantitation was done using a molar absorption coefficient of 155 mM^-1^cm^-1^. Protein concentration in the lung tissue samples was determined using the standard colorimetric BCA method with BSA as the standard. Lipid peroxidation level was calculated as nanomoles of MDA formed per milligram of protein.

### Biochemical Measurements

Activities of the anti-oxidant enzymes, Superoxide dismutase (SOD) and Catalase (CAT), were measured in the lung tissues harvested at 6 days post treatment. The SOD activity assay is based on the auto-oxidation of pyrogallol, a process highly dependent on superoxide, which is the substrate for SOD. The auto-oxidation of this compound is inhibited in the presence of SOD, whose activity was then indirectly assayed at 420 nm according to the method of Marklund and Marklund (1974). The results were represented as SOD units/mg protein.

The activity of CAT in lung tissue homogenate was assessed by measuring the initial rate of disappearance of H_2_O_2_ at 240 nm in a reaction mix containing 33 mM H_2_O_2_ in 50 mM phosphate buffer, pH 7.0 according to the method of Aebi (1984). Catalase activity in terms of μmoles hydrogen peroxide consumed per min per mg of protein was calculated using the molar extinction coefficient of hydrogen peroxide, corrected for path length. The results are represented as CAT units/mg protein.

Besides SOD and Catalase activities, the level of the non-enzymatic cellular anti-oxidant, Glutathione, was measured according to the method of Moron et al. (1979).

### Assessment of DNA Damage (Micronuclei Induction)

Micronuclei (MN) are small, cytoplasmic bodies that contain fragments of acentric/damaged chromosomes or whole chromosomes that were unable to get incorporated into the daughter nuclei during cell division. Lung tissue, harvested at 6 days post treatment, was finely minced with a scalpel and then enzymatically digested for 1 h at 37°C in a solution of 0.1% Collagenase Type IV (MP Biomedicals Inc., United States) with 2.5 mM CaCl_2_ prepared in HBSS. The resulting digest was then filtered through a 70 μm Falcon cell strainer (BD Biosciences) to remove debris and washed again with HBSS. The resulting single cell suspension was fixed with Carnoy’s fixative (3: 1 v/v, Methanol: Acetic acid) at 4°C overnight and then dropped on clean, pre-chilled microscopic glass slides. Following air drying, slides were stained with 10 μg/ml of the DNA binding fluorescent dye 4′,6-Diamidine-2′-phenylindole dihydrochloride (DAPI) (Sigma-Aldrich, United States) in phosphate buffer (0.05M Na_2_HPO_4_⋅2H_2_O, 0.05%, Tween-20, 0.01 M citric acid, pH 7.4) in dark, at room temperature for 15 min (Dwarakanath and Jain, 1985). After staining, slides were rinsed with PBS, mounted in an aqueous mounting medium (1:1 v/v, PBS-Glycerol) and observed under a fluorescence microscope (Olympus IX51, Japan) using a UV excitation filter. A total of 1000 cells were scored per group. M-fraction (MF), i.e., the percentage of cells with micronuclei, was then calculated as:

$$MF(\%)=N_{m}/N_{t}\times 100,$$

Where, N_m_ is the number of cells with micronuclei, N_t_ is the total number of cells analyzed.

### Assessment of Lung Edema

The ratio of lung wet-to-dry weight was used as an index of pulmonary edema during bleomycin injury. After euthanizing mice at 06 days post treatment, the lungs were carefully removed surgically, rinsed in PBS, blotted dry, and weighed immediately (wet weight). The same lung tissue was then placed in an oven at 60°C for 72 h and reweighed to get the dry weight. The ratio of weight-to-dry was then calculated for each animal to assess tissue edema.

### Collection of BALF

After euthanizing with chloroform, an oblique incision was made in the trachea and cannulated with a 20-gauge i.v. catheter, which was tied into place. Left lung was tied with a nylon thread and only the right lung was lavaged. 0.5 mL of cold PBS (pH 7.4) was instilled and gently aspirated three times and pooled to collect the BALF. Typically, total fluid recovery exceeded 80%. The BALF thus obtained was placed immediately on ice until processing.

### Analysis of Cell Counts and Protein Concentration in BALF

BAL samples were centrifuged at 1,500 g for 10 min at 4°C and the cell free BAL supernatant thus obtained was stored at -80°C for subsequent studies. Furthermore, the cell pellet was resuspended in PBS, and subsequently, the number of total leukocytes was determined using a hemocytometer by staining them with Turk’s fluid. A small volume of the BAL fluid was smeared on a clean glass slide for calculating the differential leukocyte count. Smears were air dried, fixed in absolute methanol for 5 min and then visualized after May Grünwald-Giemsa staining for identification of PMNs for differential leukocyte count. Total protein concentration in the BALF was determined using a standard commercial BCA kit available from Pierce (Rockford, IL, United States) using BSA as the standard.

### Determination of MPO Activity

To obtain a quantitative measure of leukocyte influx at 06 days post treatment, MPO levels were assessed in the frozen lung specimens using the Myeloperoxidase assay kit (Sigma-Aldrich, Saint Louis, MO, United States). Lung tissue was rapidly homogenized in 4 volumes of MPO Assay Buffer (provided in the kit) and centrifuged at 13,000 × *g* for 10 min at 4°C to remove the insoluble material. The supernatants were collected and MPO activity was measured as per the manufacturer’s instructions. One unit of MPO activity is defined as the amount of enzyme that hydrolyzes the substrate and generates taurine chloramine to consume 1.0 μmole of the chromophore TNB per minute at 25°C. MPO activity was calculated as milliunit/mL using the TNB standard curve and reported as fold change over control.

### Measurement of Cytokine Levels

The levels of Tumor Necrosis Factor Alpha (TNF-α), Interleukin (IL) -1β, IL-6 and Transforming Growth Factor beta (TGF-β) were quantified in clarified BALF using commercially available sandwich ELISA kits from eBioscience (San Diego, CA, United States) according to the manufacturer’s instructions.

### Western Blot Analysis

Frozen lung tissue was weighed and homogenized on ice with a micro pestle using ice cold radioimmunoprecipitation assay (RIPA) buffer. Samples were incubated on ice for 40 min., centrifuged at 10,000 g for 20 min at 4°C and supernatants collected. Following determination of the protein concentration with BCA protein assay kit (Thermo Scientific), equal quantities of protein samples (40 μg) were separated on SDS-PAGE after denaturation, transferred onto polyvinylidene difluoride (PVDF) membranes (MDI, United States), and blocked with 5% (w/v) BSA for 1 h at room temperature. The membranes were then incubated overnight at 4°C with primary antibodies against pERK1/2, ERK, p-p38, p38, COX-2 (1: 2000, Cell Signaling Technology, United States), 3-NT, α-SMA (Abcam, Boston, MA, United States) and NF-κB p65 subunit (1:1000, Santa Cruz Biotechnology, Santa Cruz, CA, United States) followed by incubation with the corresponding horseradish peroxidase conjugated secondary antibodies (1:10,000, Santa Cruz Biotechnology, Santa Cruz, CA, United States) for 2 h at room temperature. Equal protein loading was verified by re-probing of membranes with anti-β-actin antibody. Finally, the blots were visualized using Luminata^TM^ Forte Western HRP Substrate for band detection. The chemiluminescence signal was captured using Microchemi (DNR Bioimaging Systems, Israel). For densitometrical analysis of bands, the densitometric signal for the target protein was normalized to β-actin using Image J tool.

### Statistical Analysis

Data was analyzed using Graph Pad Prism (version 5.0) and the experimental results expressed as mean ± SEM. One-way or two-way ANOVA followed by the Bonferroni *post hoc* multiple comparison test was used to test the significance of any differences between groups. Results were considered significant at *p* < 0.05.

## Results

### DRDE-30 Reduced Bleomycin-Induced Histopathological and Fibrotic Changes in the Lung

**FIGURE 2:**
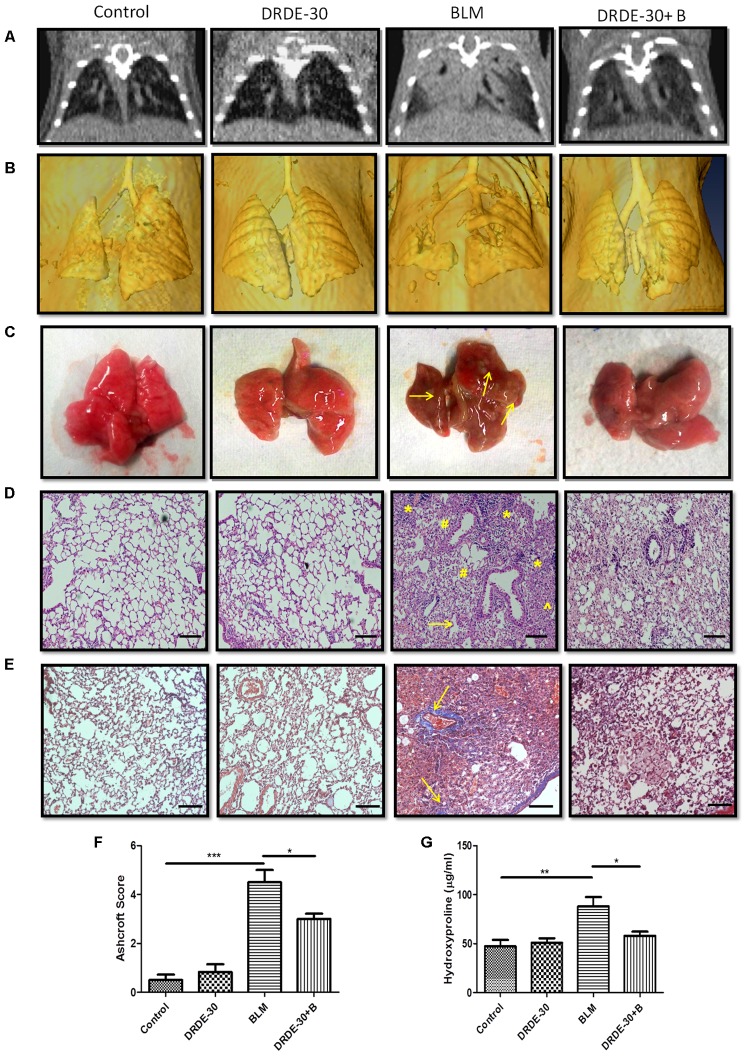
Morphological, histopathological and fibrotic changes in the lungs of mice post bleomycin injury. **(A)** Representative images of axial slices of reconstructed anatomical X-ray micro-CT images obtained at 21 days post treatment. Bleomycin treated mice show parenchymal opacity in the right lung. **(B)** Three-dimensional surface-rendered images of the lungs showing changes in the lung density between different treatment groups. **(C)** Representative images showing gross lung morphology of mice. **(D)** Representative photomicrographs of hematoxylin and eosin stained lung sections. Scale bar: 100 μm. Bleomycin treated mice developed lungs that had thickened alveolar septae (arrowhead) collapsed alveolar spaces (#) and infiltration of a large number of inflammatory cells (^∗^) in the peribroncheolar region. **(E)** Representative images of Masson’s trichrome stained lung sections at 21 days post treatment. Scale bar = 100 μm. **(F)** Assessment of pulmonary injury by Ashcroft score. Data represents mean ± SEM; *n* = 4 per group. **(G)** Hydroxyproline content, an indicator of collagen deposition, at 21 days post treatment. Data represents mean ± SEM of two independent experiments with *n* = 3–5 per group. ^∗^*p* < 0.05; ^∗∗^*p* < 0.01;^∗∗∗^*p* < 0.001.

X-ray-CT offers a rapid, non-invasive and painless method for detecting and assessing lung injuries and is widely used in thoracic radiotherapy patients to monitor its progression and treatment. Micro-CT analysis of bleomycin treated mice at day 21 showed a marked change in the lung density as compared to control mice, indicated by increased parenchymal opacity (**Figures 2A,B**). Mice pre-treated with DRDE-30 had improved lung architecture than bleomycin alone treated mice, suggestive of reduced collagen deposition and obliteration of air spaces. Mice receiving DRDE-30 alone had no observable effect on the lungs as compared to the lungs of the control mice.

Morphologically, bleomycin treated animals showed collapsed and haemorrhagic lungs with rough surfaces and white/gray fibrous nodule development. DRDE-30 pre-treated animals, on the other hand, had improved lung morphology, with no lung collapse and lesser fibrous nodules (**Figure 2C**).

Observations made during CT scan of mice corroborated well with the histo-pathological findings. At day 21 after bleomycin treatment, mice showed histological evidence of lung injury like disruption of alveolar lining, thickening of alveolar wall, loss of air spaces, in association with oedema and infiltration of inflammatory cells. Pre treatment with DRDE-30 reduced the severity of some of the bleomycin-induced anatomical changes in the lung including less oedematous changes, lesser infiltration of cells and larger air spaces, indicating that DRDE-30 was able to preserve the lung microstructure from bleomycin- induced damage (**Figure 2D**). No pathological alteration in lung architecture was observed in the animals of DRDE-30 only group. To assess the ability of DRDE-30 in minimizing the fibrotic response after bleomycin exposure, paraffin-embedded transverse sections of 5 μm were cut and stained using Masson’s trichrome to identify the sites of collagen deposition. The major constituent of collagen, Hydroxyproline (Starcher et al., 1978), was also measured in the lungs of experimental mice to correlate the findings. Bleomycin treated group displayed an increased collagen deposition, which was evident from an increase in the intensity of the blue color in the Trichrome stained lung sections. DRDE-30 treatment resulted in lesser collagen deposition and formation of fibrotic foci when compared to bleomycin treated animals, along with reduced lung pathology as evidenced by minimal structural damage (**Figure 2E**). These foci represent discrete areas consisting of actively proliferating fibroblasts, collagen-producing myofibroblasts, and newly synthesized collagen and appear as scattered, pink colored patchy areas in the lungs. These lesions are considered to be focal areas of active, ongoing lung injury that gradually progress toward fibrosis and represent the transition phase between the normal and the fibrotic lung (Hay et al., 1991; Mouratis and Aidinis, 2011). Furthermore, the severity of fibrosis was assessed semi-quantitatively using the Ashcroft score. There was a statistically significant (4.53 ± 0.51, *p* < 0.001) increase in the Ashcroft score in bleomycin administered mice, while protection of lung microstructure by DRDE-30 was clearly reflected in the lower (3.14 ± 0.22, *p* < 0.05) lung injury scores in drug-bleomycin treated mice (**Figure 2F**). In accordance with these observations, the hydroxyproline content was also found to be remarkably increased (88.1 ± 9.56 vs. Control 47.25 ± 6.58, *p* < 0.01) at 21 days after bleomycin exposure, while DRDE-30 pre-treatment had significantly (58.04 ± 4.16, *p* < 0.05) lowered the hydroxyproline levels in the lungs of bleomycin exposed animals (**Figure 2G**).

### DRDE-30 Alleviated Bleomycin-Induced Oxidative Stress and Cytogenetic Damage

Highly reactive free radicals, ROS and RNS, generated after bleomycin exposure cause macromolecular damage in the lung cells and are important contributing factors for acute lung injury (ALI) (Hay et al., 1991). Thus, modulation of lipid peroxidation and protein nitrosylation, indicators of oxidative-nitrative stress induced injury, as well as the endogenous antioxidant defense system by DRDE-30 following bleomycin exposure were assessed.

**FIGURE 3:**
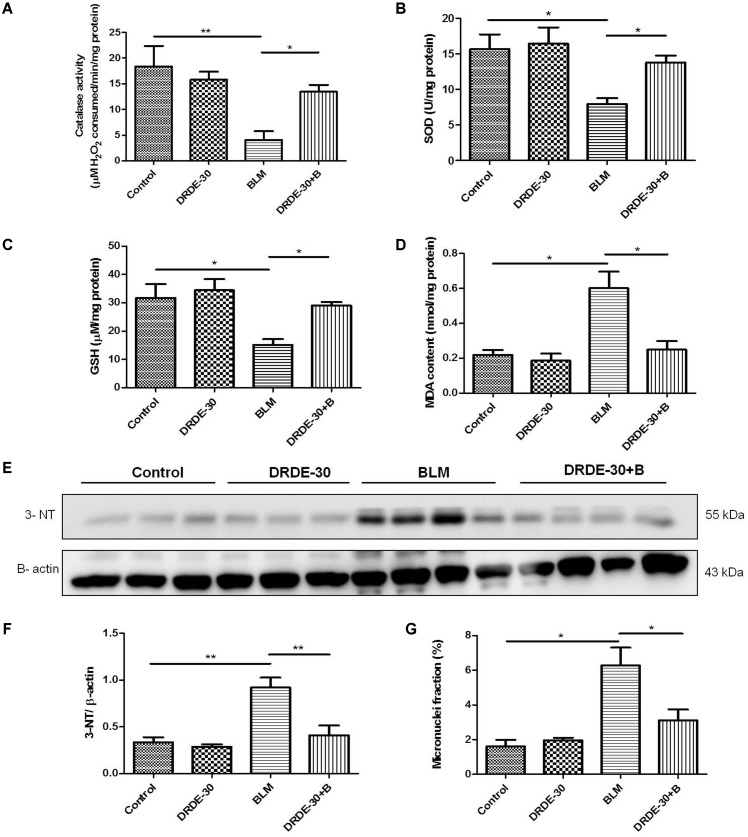
Effect of DRDE-30 on bleomycin-induced oxidative-nitrative stress and cytogenetic damage. **(A)** Catalase activity. **(B)** SOD activity. **(C)** GSH level in the lung tissues of mice at day 06 post treatment. **(D)** MDA content in the lungs of mice at 06 days post treatment. **(E)** Expression level of 3-Nitrotyrosine in the lungs of mice as assessed by western blotting at 06 days post treatment. **(F)** Densitometric quantification after normalizing with β-actin. Results are expressed as mean ± SEM. **(G)** Micronuclei induction in lung tissues of mice at day 06 post treatment. Data represents mean ± SEM of two independent experiments with *n* = 3–5 per group per experiment. ^∗^*p* < 0.05; ^∗∗^*p* < 0.01.

Bleomycin caused a notable decrease in lung SOD (*p* < 0.05) and Catalase activities (*p* < 0.01), which DRDE-30 was able to restore remarkably (*p* < 0.05) (**Figures 3A,B**). Additionally, DRDE-30 administration also enhanced the levels of the cellular anti-oxidant, GSH, which had plummeted following bleomycin challenge (29.01 ± 1.26 vs. BLM 15.2 ± 1.99, *p* < 0.05) (**Figure 3C**). Bleomycin significantly elevated the level of MDA, the final product of polyunsaturated fatty acid peroxidation (0.6 ± 0.09 vs. Control 0.22 ± 0.03, *p* < 0.05), which was inhibited by DRDE-30 pre-treatment (0.25 ± 0.05, *p* < 0.05) (**Figure 3D**). Immunoblotting of nitrotyrosine in lung tissues of mice was used to gauge the extent of nitrative stress due to peroxynitrite radicals generated during the inflammatory reaction (Chen et al., 2003). There was a marked increase in the expression of nitrotyrosine (*p* < 0.01) in the lungs, which was reduced by DRDE-30 in response to bleomycin injury (*p* < 0.05) (**Figures 3E,F**). These results revealed that DRDE-30 suppresses the deleterious consequences of oxidative reaction caused by bleomycin in mouse lungs by potentiating the lung’s anti-oxidant defense mechanisms.

Besides generation of reactive species, which are partially responsible for its toxic effects, bleomycin also causes DNA damage through DNA strand scission, leading to cytogenetic damage in the form of chromosome aberrations that manifests as micronuclei in the post-mitotic daughter cells. Micronuclei are thus indicators of genotoxic events. The percentage of lung cells with micronuclei was nearly fourfolds higher in mice exposed to bleomycin (6.28 ± 1.02 vs. Control 1.61 ± 0.39, *p* < 0.05), which was reduced by nearly 50% with DRDE-30 pre-treatment (3.11 ± 0.63, *p* < 0.05) (**Figure 3G**).

### DRDE-30 Reduced Bleomycin-Induced Vascular Leak

Early bleomycin damage to the lung is characterized by damage to the endothelial lining of small vessels and capillaries, accompanied by vascular congestion and increased microvascular permeability (Hay et al., 1991), leading to an inflammatory response. The effect of DRDE-30 on bleomycin-induced lung vascular leak was assessed by analyzing the lung wet/ dry weight ratio, total and differential leukocyte count and total protein content in BALF.

**FIGURE 4:**
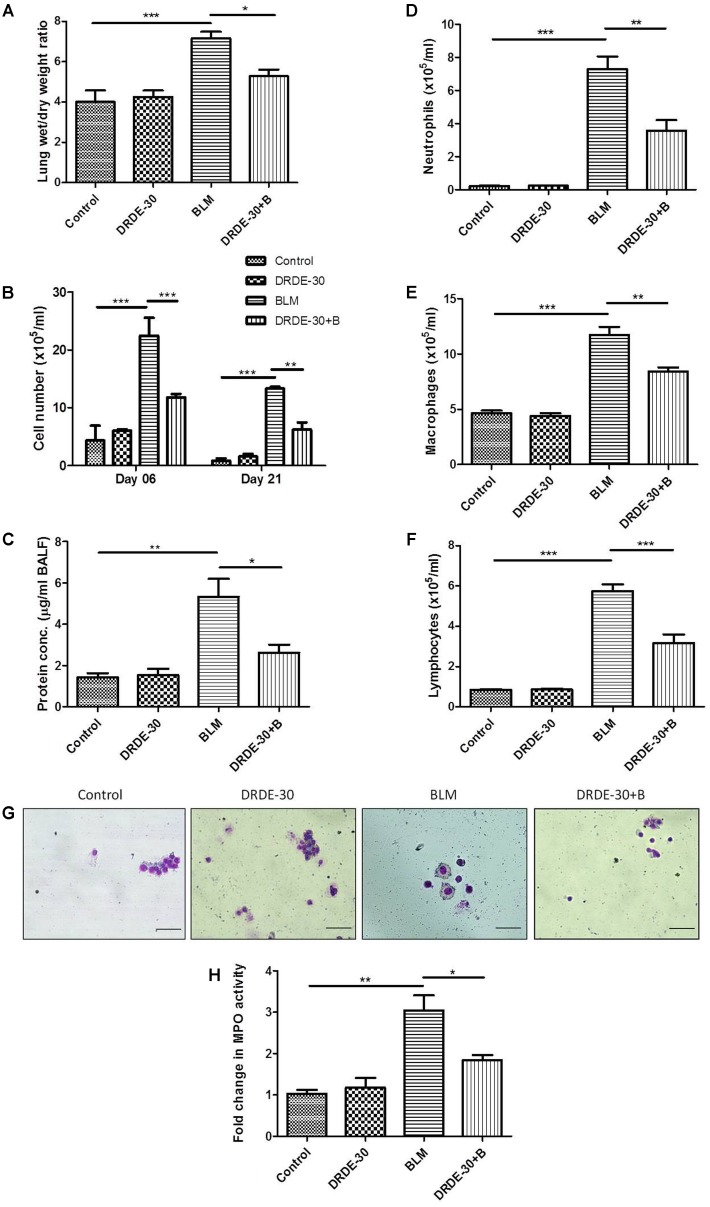
Effect of DRDE-30 on vascular leak and cellular composition of BALF following bleomycin injury. **(A)** Lung wet to dry weight ratio at 06 days post treatment. **(B)** Temporal changes in the total leukocyte count in BAL fluid, harvested at various times post treatment. **(C)** Total protein in BALF at 06 days post treatment. **(D)** Neutrophil, **(E)** Macrophage, and **(F)** Lymphocyte counts in BALF. **(G)** Representative photomicrographs of May-Grünwald-Giemsa stained BALF cells at 06 days post treatment. Scale bar: 50 μm. **(H)** Fold change in MPO activity in lungs at 06 days post treatment. Data represents mean ± SEM of two independent experiments with *n* = 3–5 per group per experiment. ^∗^*p* < 0.05; ^∗∗^*p* < 0.01; ^∗∗∗^*p* < 0.001.

Lung fluid accumulation, a reflection of pulmonary oedema, measured by calculating the ratio of lung wet to dry weight at day 06, was pronounced in bleomycin treated animals (7.15 ± 0.33 vs. Control 4.03 ± 0.58, *p* < 0.001), which was blunted by pre administration of DRDE-30 (5.28 ± 0.31, *p* < 0.05) (**Figure 4A**).

An increase in BAL cellularity was observed at all the time points studied (day 06, day 21) in bleomycin treated (*p* < 0.001) as compared to the untreated control mice (**Figure 4B** and **Table 1**), which was in line with the increased infiltration of inflammatory cells observed in the histology (**Figure 2D**). The increase in cellular infiltration was accompanied by a concomitant increase in protein leakage in lungs. On day 06, total BAL protein was significantly elevated in bleomycin alone group (5.33 ± 0.87 vs. Control 1.44 ± 0.19, *p* < 0.01). In contrast, DRDE-30 showed a protective effect and reduced the total amount of protein leaked into the lung (2.63 ± 0.39, *p* < 0.05) (**Figure 4C**).

**Table 1:** Effect of DRDE-30 on bleomycin-induced changes in total and differential cell counts in the broncheoalveolar lavage fluid of mice.

Group	Total cells (×10^5^/ml) Day 06	Total cells (×10^5^/ml) Day 21	Neutrophils (×10^5^/ml) Day 06	Macrophages (×10^5^/ml) Day 06	Lymphocytes (×10^5^/ml) Day 06
Control	4.35 ± 2.56	0.85 ± 0.43	0.2259 ± 0.02	4.656 ± 0.23	0.8469 ± 0.04
DRDE-30	6.03 ± 0.26	1.59 ± 0.42	0.2338 ± 0.03	4.404 ± 0.24	0.8649 ± 0.04
BLM	22.47 ± 3.09^∗∗∗^	13.33 ± 0.3^∗∗∗^	7.297 ± 0.76^∗∗∗^	11.73 ± 0.72^∗∗∗^	5.733 ± 0.35^∗∗∗^
DRDE-30+B	11.80 ± 0.6^###^	6.23 ± 1.25^##^	3.561 ± 0.65^##^	8.436 ± 0.34^##^	3.178 ± 0.42^###^


*Data represents mean ± SEM of two independent experiments with *n* = 3–5 per group per experiment. ^∗∗∗^*p* < 0.001 compared to control; ^##^*p* < 0.01 and ^###^*p* < 0.001 compared to BLM. BLM, Bleomycin.*

The cellular infiltrate consisted predominantly of macrophages (*p* < 0.001), followed by neutrophils (*p* < 0.001), and lymphocytes (*p* < 0.001) (**Figures 4D–G** and **Table 1**). Increased neutrophil infiltration was reflected in a commensurate threefold increase in the MPO activity in lung (*p* < 0.01) (**Figure 4H**). Pre-treatment with DRDE-30 significantly reversed these pathological changes as reflected in the near restoration of all parameters studied to the untreated values.

### DRDE-30 Attenuated Bleomycin-Induced Inflammatory Response

**FIGURE 5:**
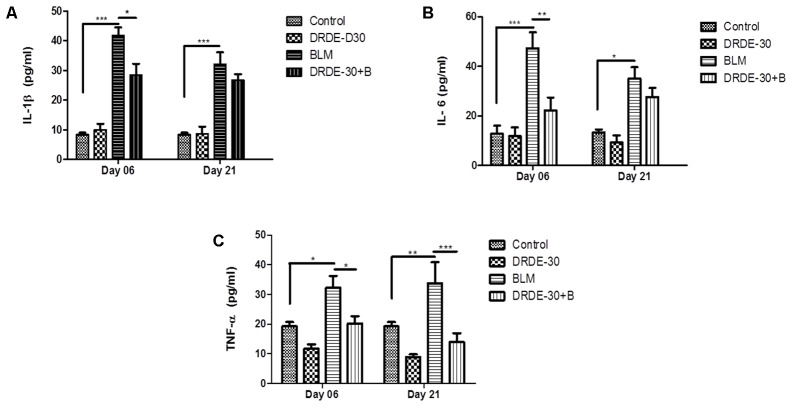
Effect of DRDE-30 on secretion levels of pro-inflammatory cytokines. **(A)** IL-1β, **(B)** IL-6, and **(C)** TNF-α levels in BALF harvested at various times post treatment. Data represents mean ± SEM; *n* = 6 per group. ^∗^*p* < 0.05; ^∗∗^*p* < 0.01; ^∗∗∗^*p* < 0.001.

Early inflammatory response after bleomycin treatment involves the release of pro-inflammatory cytokines, which promote the chemotaxis of neutrophils and macrophages into the lung tissue by creating a positive feedback loop (Moeller et al., 2008). The BALF levels of IL-1β and IL-6 decreased temporally after bleomycin treatment, with the maximal levels at day 06 post exposure (*p* < 0.001), though still being significantly higher than the control group at both the time points studied (**Figures 5A,B**). TNF-α levels were also elevated in the bleomycin alone treated animals at both day 06 (*p* < 0.05) and day 21 (*p* < 0.01) post exposure as compared to the control group (**Figure 5C**). The augmentation in the secretion levels of all the three studied cytokines was significantly abated by DRDE-30 pre-treatment at day 06 of treatment. However, there was marginal, though not significant reduction in the levels of BALF IL-1β and IL-6 at day 21. In contrast, the levels of TNF-α were markedly reduced even at day 21 after DRDE-30 treatment (*p* < 0.001).

### DRDE-30 Suppressed the Activation of Inflammatory Signaling

**FIGURE 6:**
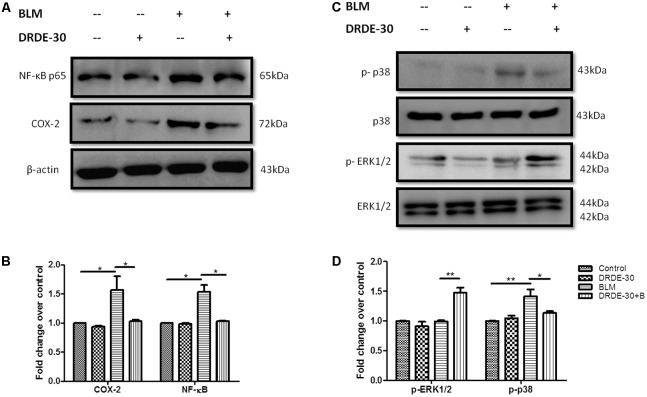
Effect of DRDE-30 on bleomycin-induced changes in inflammatory and redox-sensitive signaling mediators. **(A)** Changes in the protein expression of NF-κB and COX-2. **(B)** Densitometry of target proteins after normalizing with β-actin. Results are expressed as mean ± SEM (*n* = 3 per group). **(C)** Molecular status of phosphorylated ERK and p38 at 06 days post infliction of bleomycin injury. **(D)** Densitometry of target proteins after normalizing with β-actin. Results are expressed as mean ± SEM (*n* = 2 per group). ^∗^*p* < 0.05; ^∗∗^*p* < 0.01.

Bleomycin-induced oxidative stress and DNA damage has been shown to activate various signaling molecules, such as p38 and JNK MAP kinases, which are closely involved in the control of vascular permeability, inflammation and connective tissue remodeling through induction of NF-κB along with enzymes such as Cyclooxygenase (COX-2) (Matsuoka et al., 2002; Singer et al., 2003). Western blot analysis for studying the modulatory effects of DRDE-30 on p38 phosphorylation, COX-2 and NF-κB activation in response to bleomycin challenge revealed that DRDE-30 significantly suppressed the activation of these inflammatory mediators post induction of bleomycin injury (*p* < 0.05) (**Figures 6A,B**). Since oxidative stress is one of the stimuli for activation of the p38 MAPK signaling cascade, restoration of the anti-oxidant enzyme levels by DRDE-30 partly accounts for the reduced level of p38 phosphorylation. Also, since the inflammatory state developed after bleomycin treatment is linked to ROS and release of pro-inflammatory cytokines; it also suggests the potent inhibitory effect of DRDE-30 on the associated inflammatory pathways responsible for these effects.

Activation of the ERK1/2 MAPK signaling cascade has been associated with suppression of apoptosis in response to a variety of stimuli; one of them being TNF- α (Tran et al., 2001; Allan et al., 2003). Since it has been shown that the lung epithelial cells sustain DNA damage and undergo apoptosis during PF (Kuwano et al., 1996; Barbas-Filho et al., 2001), we assessed the effect of DRDE-30 on the activation of the ERK/ MAPK pathway. Indeed, DRDE-30 mediated a 1.5 fold increase in the level of phosphorylated ERK1/2 in response to bleomycin treatment (*p* < 0.01) (**Figures 6C,D**), thereby exerting a pro-survival effect, perhaps by inhibiting apoptosis in lung alveolar cells and reducing the inflammatory effects of bleomycin. Since, it also overcomes the oxidative stress induced by bleomycin by strengthening the cellular anti-oxidant defense system; ERK1/2 activation could be an important underlying mechanism behind the protective effects of DRDE-30.

### DRDE-30 Abrogated the Expression of Fibrotic Mediators

TGF-β is an important pro-fibrogenic cytokine that regulates the process of EMT during fibrosis (Willis and Borok, 2007). We, thus, examined the effect of DRDE-30 on these two fibrotic mediators.

**FIGURE 7:**
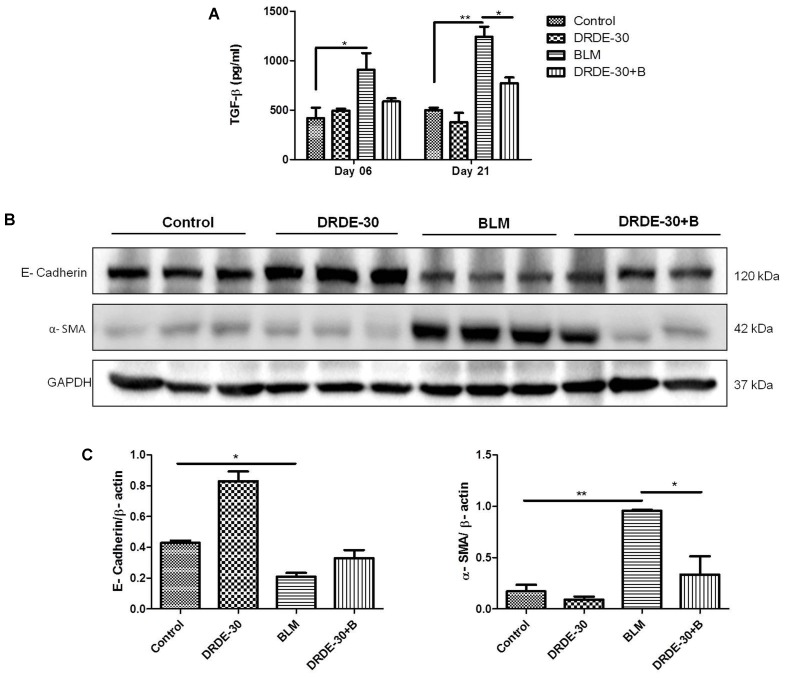
Effect of DRDE-30 on bleomycin-induced changes in key fibrotic mediators. **(A)** BALF levels of the pro-fibrotic cytokine, TGF-β. Data represents mean ± SEM, *n* = 6 per group. **(B)** Changes in the protein expression of the EMT markers *E*-Cadherin and α-SMA as assessed by western blotting at 21 days post treatment. **(C)** Densitometry of target proteins after normalizing with β-actin. Results are expressed as mean ± SEM. ^∗^*p* < 0.05; ^∗∗^*p* < 0.01; ^∗∗∗^*p* < 0.001.

The BALF TGF-β level rose gradually (908.48 ± 166.82 vs. Control 417.89 ± 106.88, *p* < 0.05 at day 6) and was maximally elevated at day 21 post bleomycin treatment (1243.57 ± 99.36 vs. Control 500.02 ± 24.3, *p* < 0.01). The degree of increase was reduced in DRDE-30 pre-treated mice and a marked reduction was observed only at day 21 after treatment (772.69 ± 56.90, *p* < 0.05) (**Figure 7A**). The decrease in TGF-β levels could be attributed to the heightened anti-oxidant defense system leading to a diminished expression of the pro-fibrogenic cytokine, responsible for the proliferation and activation of myofibroblasts which synthesize collagen. One of the mechanisms leading to the proliferation and activation of myofibroblasts is EMT (Thiery et al., 2009). DRDE-30 pre-treatment did not reduce the level of the epithelial marker E-Cadherin significantly, but led to a markedly abrogated expression of the mesenchymal marker α-SMA in the lungs of mice, indicative of its subduing effect on EMT (**Figures 7B,C**). These findings gain support from our observation of decreased TGF-β levels in DRDE-30/BLM treated mice, since TGF-β is one of the important factors that influence the process of EMT.

## Discussion

The present study was undertaken to evaluate the protective effects of DRDE-30 in an animal model of pulmonary fibrosis induced by intra-tracheal instillation of bleomycin. This model has contributed tremendously in elucidating the important roles played by cytokines, growth factors and signaling pathways in pulmonary fibrosis (Kalayarasan et al., 2008; Matsuoka et al., 2002), besides identifying TGF-β as one of the key pro-fibrogenic factors (Willis and Borok, 2007) The choice of using female C57BL/6 mice as the model system was based on their reported higher susceptibility to bleomycin-induced fibrosis compared to other strains like BALB/c mice, which are relatively fibrosis-resistant, attributable to the differential expression of various cytokines and proteases/anti-proteases (Schrier et al., 1983; Phan and Kunkel, 1992). Additionally, female mice have been shown to have an exaggerated response to bleomycin-induced lung injury than their male counterparts due to the female sex hormones that have a direct pro-fibrogenic effect on the lung fibroblasts (Gharaee-Kermani et al., 2005). We employed micro-CT, a form of non-invasive imaging modalities that provides high-resolution anatomical images of small animals. Further, it allows repeated measurements in individual animals, allowing the visualization of disease progression or reversibility due to interventions (Coxson, 2007), thereby reducing the need for large number of animals, and avoids animal to animal variations confronted with invasive methods that use euthanasia. Micro-CT was performed at 21 days of treatment as work done previously with bleomycin in mouse models has shown that full blown fibrosis develops by 21–28 days after bleomycin administration (Moeller et al., 2008; Walters and Kleeberger, 2008; Liu et al., 2017). Indeed, micro-CT unequivocally revealed the protective effects of DRDE-30 against BLM-induced lung injury, visualized as changes in the lung density due to differential attenuation of X-rays by fibrotic areas in comparison to normal lung parenchyma (**Figures 2A,B**).

**FIGURE 8:**
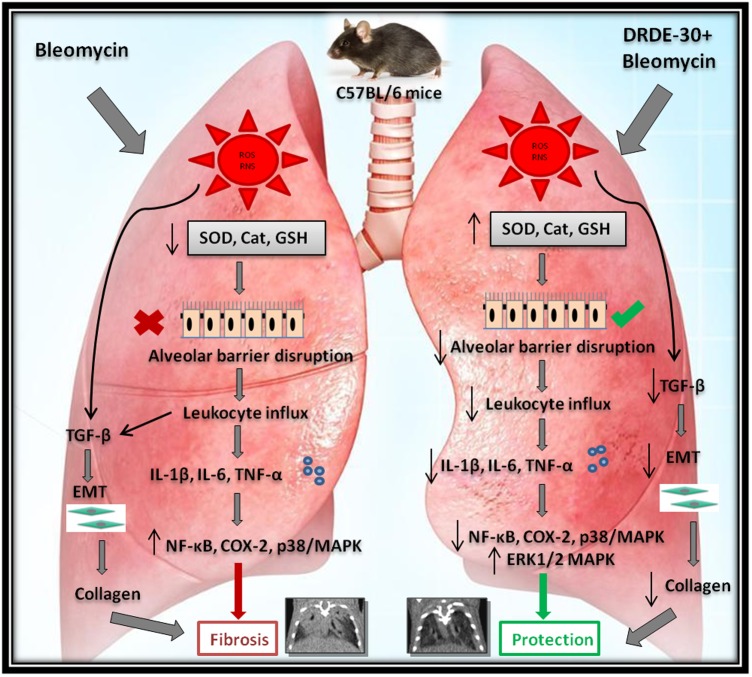
Diagrammatic representation of the plausible mechanisms by which DRDE-30 confers protection against bleomycin-induced pulmonary injury. Bleomycin-induced lung injury is initiated with the production of reactive intermediates causing oxidative stress, and subsequent damage to the endothelial and the epithelial cells. This leads to the disruption of the barrier between the alveolar and the capillary network, causing infiltration of inflammatory cells into the lung tissue. Release of inflammatory mediators and pro-fibrogenic cytokines by the immune cells promotes proliferation of myofibroblasts, collagen deposition and ultimately organ failure, which may culminate into death of the organism. DRDE-30 provides protection against bleomycin-induced lung damage by activating anti-oxidant pathways and abrogating the inflammatory and fibrotic changes.

Bleomycin induces a complex response in the lung, one which occurs in several distinct phases, with the fundamental event being generation of reactive radical species by iron dependent mechanism. Increased amounts of oxygen and nitrogen radicals generated by the resident and the migrated immune cells aggravate the damage, augmenting inflammation and causing cytokine dysregulation, culminating into fibrosis (Adamson and Bowden, 1974; Hay et al., 1991; Izbicki et al., 2002). MDA, formed as a result of peroxidation of cell membrane lipids by oxygen-derived free radicals, is considered a reliable marker of oxidative stress induced tissue damage (Torun et al., 2009). The increased oxidative (MDA) and nitrative (3-NT) damage observed in bleomycin challenged mice (**Figures 3D–F**) could be due to free radical mediated damage to membranes and proteins, since overproduction of reactive intermediates has an inhibitory effect on the enzymatic and non-enzymatic antioxidant defense systems (Blum and Fridovich, 1985; Sogut et al., 2004). Concentrations of GSH usually present in the epithelial lining can curb mitogen-induced fibroblast proliferation, thus implicating the role of an insufficiency of GSH in the pathogenesis of pulmonary fibrosis (Cantin et al., 1989). A notable descend in GSH level observed in bleomycin-challenged animals (**Figure 3C**) appears to be due to the utilization of this antioxidant to counter the overproduction of free radicals (Blum and Fridovich, 1985). Administration of DRDE-30 reduced bleomycin-induced oxidative stress and activation of redox-sensitive signaling cascades, accompanied by a reduction in the infiltration of inflammatory cells, release of pro-inflammatory cytokines and excessive collagen deposition in mouse lung tissues (**Figure 8**). Since oxidative stress could indeed drive these processes of tissue damage, these observations suggest that the ability of DRDE-30 to reduce bleomycin-induced oxidative stress may be primarily responsible for the reduction in lung damage and subsequently, inflammation and fibrosis. The findings of our study gain support from the studies done earlier with antioxidant agents in bleomycin lung injury model, which had demonstrated their anti-inflammatory as well as anti-fibrotic effect (Felton et al., 2009; Odajima et al., 2010; Akgedik et al., 2012; Chitra et al., 2013; Verma et al., 2013; Kilic et al., 2014; Dong et al., 2017). Although numerous studies have previously reported beneficial effects of DRDE-30 against Sulfur Mustard toxicity, this study, for the first time, sheds light on the protective potential of DRDE-30 in reducing pulmonary fibrosis induced by bleomycin.

It is now recognized that bleomycin damage to the lung cells is mediated by DNA strand scission that leads to increased production of free radicals (Della Latta et al., 2015). DRDE-30 has already been demonstrated to reduce DNA fragmentation induced by Sulfur Mustard in hepatic cells (Kulkarni et al., 2006), which supports our observation of reduction in micronuclei induction by DRDE-30 in lung cells following bleomycin injury (**Figure 3G**), owing to its anti-oxidative capacity. Wet-to-dry lung weight ratio is a measure of interstitial oedema and has been used as an indirect index of acute bleomycin lung injury due to disruption of the alveolar-capillary barrier (Hay et al., 1991). We found that DRDE-30 significantly blunted bleomycin-induced vascular leak indicated by attrition in the wet-to-dry lung weight ratio, accumulation of protein and leukocyte migration into the alveolar space (**Figure 4**), suggesting that DRDE-30 plays a protective role by reducing the free radical mediated damage induction in epithelial and endothelial cells, thereby preserving the vascular-alveolar barrier, which alleviates the subsequent inflammatory and fibrotic events (**Figure 8**).

Bleomycin exposure leads to the generation of an inflammatory response elicited by overproduction of free radicals, which along with induction of pro-inflammatory cytokines and activation of immune cells, simulates acute lung injury. Pro-inflammatory cytokines such as TNF-α, IL-6 and IL-1β have been implicated in the development of fibrosis in bleomycin animal models (Piguet et al., 1989; Phan and Kunkel, 1992). TNF-α and IL-1β have been associated with airway fibrosis because of their ability to regulate fibroblast proliferation and synthesis of extracellular matrix (Elias et al., 1999). One of the major sources of these detrimental inflammatory cytokines is the macrophage. It has been shown that bleomycin activates alveolar macrophages *in vitro*, which then release pro-inflammatory cytokines such as TNF-α and IL-1β, among others (Scheule et al., 1992). Besides macrophages, neutrophils have also been documented to play role in lung parenchymal injury by releasing harmful free radicals and various proteolytic factors (Taooka et al., 1997). The initial rise in the level of pro-inflammatory cytokines (IL-1, TNF-α, IL-6, IFN-γ) mediated by increased activity of NF-κB (Koptides et al., 1997), is followed by an increase in the expression of pro-fibrotic markers (TGF-β1, fibronectin), which peak around day 14 (Chaudhary et al., 2006). We observed that the reactive oxygen and nitrogen species released following bleomycin exposure resulted in the activation of the redox-sensitive transcription factor NF-κB (**Figures 6A,B**), which regulates the transcription of many cytokines, including TNF-α, IL-1 and IL-6 and triggers inflammatory signaling (Pahl, 1999). This was in accordance with previous observations, which implicate ROS in the activation of NF-κB through phosphorylation of I-kB (Fan et al., 2003). These cytokines may in turn, also activate NF-κB, indicating that the sustained activation of this transcription factor has a critical role to play in the expression of the inflammatory mediators in this animal model. Attenuation of bleomycin-induced neutrophilia and recruitment of macrophages and lymphocytes into lungs (**Figures 4D–G**), lung MPO activity (**Figure 4H**), together with a dampened expression of NF-κB (**Figures 6A,B**), TNF-α, IL-6 and IL-1β (**Figure 5**), suggests that DRDE-30 may reduce bleomycin-induced fibrosis by limiting the activation of redox sensitive inflammatory pathways in the lung. These observations are in agreement with previous reports, which show that antioxidants can inhibit oxidant induced NF-κB activation (Gurujeyalakshmi et al., 2000).

Besides activating the NF-κB signaling pathway, ROS can cause the activation of p38/MAPK and COX-2 signaling, which are intimately involved in the control of inflammatory responses. Besides oxidative stress, the p38 family of MAP kinases can be phosphorylated and activated in response to a plethora of extracellular stimuli, including DNA damage (Raingeaud et al., 1995; Zarubin and Han, 2005), and are known to play essential roles in the induction of pro-inflammatory cytokines and enzymes such as COX-2, which orchestrate connective tissue remodelling in various pathologies (Beyaert et al., 1996; Matsuoka et al., 2002; Pini et al., 2012). The p38/MAPK are also involved in TNF-α and Fas/Fas ligand pathway mediated apoptosis of lung epithelial and endothelial cells, which is the primary cause of loss of alveolar structure during bleomycin injury (Hagimoto et al., 1997; Zhang et al., 2000). In the present investigation, DRDE-30 markedly suppressed the activation of p38/MAPK (**Figures 6C,D**) and expression of COX-2 (**Figures 6A,B**) in response to bleomycin challenge, suggestive of its potent anti-oxidant and anti-inflammatory capacity. This blocking effect of DRDE-30 might be crucial for controlling the upregulation of pro-inflammatory and fibrogenic cytokine genes during induction of lung injury by bleomycin. This also indicates that perhaps, DRDE-30 could also have an inhibitory effect on the apoptosis of lung cells, through suppression of the p38/MAPK pathway, preserving the lung architecture in response to bleomycin exposure (**Figure 2**). In addition to the inhibition of the p38/MAPK activation, DRDE-30 also led to an enhancement in the activation of ERK1/2, another member of the MAPK family, in response to bleomycin exposure (**Figures 6C,D**). ERK1/2 can afford anti-apoptotic effects by downregulating the expression of pro-apoptotic molecules and promote cell survival by upregulating anti-apoptotic molecules (Lu and Xu, 2006; Fukumoto et al., 2010). Indeed, the deleterious histo-pathological alterations induced by bleomycin in the lung were significantly reduced upon treatment with DRDE-30 (**Figure 2D**), which can be attributed to the potential anti-apoptotic and pro-survival effects of DRDE-30.

Bleomycin induced lung fibrosis is a gradual and sequential process, with epithelial cell death occurring around days 1–3 after exposure due to overproduction of ROS, followed by influx of inflammatory cells from days 3–9. In the aftermath of the explosive inflammatory response, a fibro-proliferative response follows from days 10–21, which comprises of proliferation and activation of fibroblasts into myofibroblasts, accompanied by synthesis and deposition of extra cellular matrix in the alveolar spaces, causing abnormal remodeling of alveolar parenchyma and stiffening of the lungs (Hardie et al., 2009; Tashiro et al., 2017). The transition from the initial inflammatory to the late fibrotic phase begins to occur around day 9 after bleomycin challenge, with a peak around day 14 (Chaudhary et al., 2006). The matrix secreting fibroblasts are activated by bleomycin either directly (Moseley et al., 1986) or indirectly by cytokines such as TNF-α and IL-1β, that are induced following bleomycin exposure (Schmidt et al., 1982; Sugarman et al., 1985). TGF-β, a critical pro-fibrogenic cytokine induced by ROS, has been demonstrated to contribute to a broad spectrum of events taking place during the pathogenesis of pulmonary fibrosis such as differentiation of fibroblasts into active myofibroblasts (Lee et al., 2006), synthesis and deposition of ECM molecules by myofibroblasts (Ignotz and Massagué, 1986) and EMT (Willis and Borok, 2007). The process of EMT during which the fibroblasts transform into myofibroblasts by losing epithelial cell-related markers (such as *E*-cadherin, ZO-1) and gaining mesenchymal cell-related markers (e.g., α-SMA, N-cadherin, vimentin) (Thiery et al., 2009; Gonzalez and Medici, 2014) promotes the deposition of extracellular matrix, thus contributing to the development of pulmonary fibrosis. DRDE-30 pre-treated mice exhibited reduced levels of soluble collagen as well as collagen deposited in alveolar spaces (**Figures 2E,F**), which could be attributed to the reduction in the level of TGF-β (**Figure 7A**) and α-SMA in the lungs of these mice (**Figures 7B,C**). This is indicative of reduction in fibrosis via downregulation of EMT and thus, collagen deposition, signifying the anti-fibrotic effect of DRDE-30. The observations made by micro-CT were thus, a footprint of the protective effects of DRDE-30 at the cellular level.

Taken together, the results of the present study demonstrate the potent protective effects of DRDE-30 against bleomycin-induced lung injury, mediated through the alleviation of oxidative-nitrative stress and inflammation, suppression of redox-sensitive MAP kinases, the NF-κB inflammatory cascade and other pro-fibrotic mediators (**Figure 8**). The results also suggest, for the first time, that use of DRDE-30 may be a potent preventive strategy for a wide spectrum of lung pathologies involving elevation of oxidative stress and inflammation; particularly for preventing the development of bleomycin-induced lung fibrosis during chemotherapy. This gains support from our *in vitro* studies on the chemo-modifying effect of DRDE-30 with Bleomycin, Etoposide, Paclitaxel, and Camptothecin, where DRDE-30 did not compromise the anti-tumor effects of these drugs (unpublished data). Since bleomycin is known to selectively affect lungs, eyes and skin, it would be desirable to study the pharmacokinetic, bio-distribution in particular, and safety profile of DRDE-30 to support its beneficial effects in the lung *in vivo*. Further, to unequivocally establish the protective potential of DRDE-30 in pulmonary fibrosis and establish its use in clinical settings, its effects on radiation induced lung injury also need to be investigated, since the bleomycin model still has limitations regarding understanding the progressive nature of human PF, in spite of advantages and certain similarities in development of tissue damage. The fibrotic response developed during bleomycin exposure is at least partially reversible, independent from any interventions (Izbicki et al., 2002) and the aspect of gradual and irreversible progression of PF (one of the critical hallmarks) in patients is not reproduced in the bleomycin model (Chua et al., 2005). We have recently established the efficacy of DRDE-30 in reducing the mortality of whole body irradiated mice and investigated its beneficial effects in radiation induced lung injury (unpublished data). Since DRDE-30 is itself an active compound, unlike Amifostine which is a pro-drug, it may protect different tissues alike, independent of the alkaline phosphatase levels. This, however, necessitates the investigations on its effects in normal tissues versus tumor tissue during anti-cancer therapy using bleomycin or radiation before contemplating its use as an adjuvant in cancer therapy.

## Author Contributions

AA, AB, and BD conceived and designed the experiments. AA performed the experiments with VB and PH (micro-CT). UP and SM synthesized DRDE-30. AB, BR, and RvS provided technical supervision and logistic support. AA analyzed the data and prepared the preliminary draft of the manuscript. AB, RjS, and BD edited, made critical revisions and approved the final version of the manuscript. All authors read and approved the final manuscript.

## Conflict of Interest Statement

The authors declare that the research was conducted in the absence of any commercial or financial relationships that could be construed as a potential conflict of interest.
